# The Artemisinin-Derived Autofluorescent Compound BG95 Exerts Strong Anticytomegaloviral Activity Based on a Mitochondrial Targeting Mechanism

**DOI:** 10.3390/ijms21155578

**Published:** 2020-08-04

**Authors:** Markus Wild, Friedrich Hahn, Benedikt Grau, Lars Herrmann, Aischa Niesar, Martin Schütz, Melanie M. Lorion, Lutz Ackermann, Svetlana B. Tsogoeva, Manfred Marschall

**Affiliations:** 1 Institute for Clinical and Molecular Virology, Friedrich-Alexander University of Erlangen-Nürnberg (FAU), Schlossgarten 4, 91054 Erlangen, Germany; markus.wild@uk-erlangen.de (M.W.); aischa.niesar@uk-erlangen.de (A.N.); martin.schuetz@extern.uk-erlangen.de (M.S.); 2 Institute of Organic Chemistry I, FAU, Nikolaus-Fiebiger-Straße 10, 91058 Erlangen, Germany; benedikt.grau@fau.de (B.G.); lars.herrmann@fau.de (L.H.); svetlana.tsogoeva@fau.de (S.B.T.); 3 Institute for Organic and Biomolecular Chemistry, Georg-August-Universität Göttingen, Tammannstraße 2, 37077 Göttingen, Germany; melanie.lorion@chemie.uni-goettingen.de (M.M.L.); lutz.ackermann@chemie.uni-goettingen.de (L.A.)

**Keywords:** human cytomegalovirus, antiviral drugs, artemisinin derivative, autofluorescence, intracellular tracking, mitochondrial impairment, mitochondrial targeting, mitochondrial architecture

## Abstract

Human cytomegalovirus (HCMV) is a major human pathogen associated with severe pathology. Current options of antiviral therapy only partly satisfy the needs of a well-tolerated long-term treatment/prophylaxis free from drug-induced viral resistance. Recently, we reported the strong antiviral properties in vitro and in vivo of the broad-spectrum anti-infective drug artesunate and its optimized derivatives. NF-κB signaling was described as a targeting mechanism and additional target proteins have recently been identified. Here, we analyzed the autofluorescent hybrid compound BG95, which could be utilized for intracellular visualization by confocal imaging and a tracking analysis in virus-infected primary human fibroblasts. As an important finding, BG95 accumulated in mitochondria visualized by anti-prohibitin and MitoTracker staining, and induced statistically significant changes of mitochondrial morphology, distinct from those induced by HCMV infection. Notably, mitochondrial membrane potential was found substantially reduced by BG95, an effect apparently counteracting efficient HCMV replication, which requires active mitochondria and upregulated energy levels. This finding was consistent with binding properties of artesunate-like compounds to mitochondrial proteins and thereby suggested a new mechanistic aspect. Combined, the present study underlines an important role of mitochondria in the multifaceted, host-directed antiviral mechanism of this drug class, postulating a new mitochondria-specific mode of protein targeting.

## 1. Introduction

Human cytomegalovirus (HCMV), the prototype species of subfamily *Betaherpesvirinae*, is a major human pathogen with global distribution. Seropositivity rates in the adult human population range between 40% and 95% depending on socio-geographic factors. HCMV infection presents a challenging clinical problem in stem cell/organ transplantation, antitumoral chemotherapy, immunodeficiency, and particularly during pregnancy in the setting of congenital infection of the unborn. As HCMV infection is associated with severe symptoms including embryonal developmental defects or life-threatening disease, improved preventive measures are needed [1,2]. Approximately 10% of infants with congenital infection display symptoms at birth, which includes microcephaly, intracranial calcifications, or even a severe, generalized cytomegalic inclusion disease [3,4]. In further 10–15%, late-onset HCMV pathology is seen at later time points. So far, no HCMV vaccine has been licensed. Antiviral therapy is mainly based on inhibitors of viral DNA synthesis such as ganciclovir (GCV), valganciclovir (VGCV), foscarnet (FOS), and cidofovir (CDV), all of which face limitations concerning therapeutic compatibility and induction of drug-resistant HCMV variants [5]. A recently approved anti-HCMV drug, terminase inhibitor letermovir (Prevymis^®^), is successfully used in the prophylaxis of HCMV disease post-transplantation [6,7,8,9,10,11,12]. However, the problem of selecting drug-resistant viruses arises even with this very potent compound [8,13], so that novel antiviral drug candidates are still the goal of international investigations.

Artemisinin, its semisynthetic derivative artesunate (ART) and related chemical compounds represent a promising antiherpesviral lead compound scaffold, which has intensively been investigated by our research group [14,15,16,17,18,19,20,21,22,23,24,25,26,27] and by other researchers [28,29,30,31,32,33,34,35,36,37,38,39]. While the original drug artemisinin proved to be poor in anticytomegaloviral activity, its semisynthetic derivative ART possessed a relatively broad antiviral activity that extended over HCMV to other herpesviruses and even human pathogenic viruses of other families [17,23,24,40,41,42,43,44,45,46,47]. The very promising potential of the entire group of artemisinin compounds (a chemical class of trioxanes/sesquiterpenes) has been confirmed by an increase in antiviral efficacy when applying the chemical hybridization concept [18,19,48]. In particular, the trimeric derivative TF27 and related compounds exert pronounced anti-HCMV activity in the submicromolar to nanomolar range both in vitro and in vivo [27,49,50,51].

As far as the antiviral mode of action of artemisinin-derived compounds is concerned, we and others previously reported on signaling effects. NF-κB-dependent signaling was shown to be modulated by artesunate and related compounds, with an inhibitory activity specifically focused on the main target NF-κB RelA/p65 [16,27,52,53,54,55,56,57]. Interestingly, an inhibitory effect of ART and related compounds has already been demonstrated on the HCMV immediate-early gene expression and the pre-incubation of compounds before HCMV infection increases the inhibitory efficacy [15,16]. It should be stressed, however, that additional modes of activity and putative protein targets have strongly been suggested by recent reports [58,59,60,61]. In the present study, we investigated the artemisinin-derived compound BG95, which exerts a strong anticytomegaloviral activity in combination with autofluorescent properties, allowing for intracellular tracking analyses. Notably, BG95 showed a specific mitochondrial accumulation, led to dose-dependent changes of mitochondrial architecture, and reduced mitochondrial membrane potential in HCMV-infected cells. A refined view of the postulated multifaceted antiviral action of BG95, also based on a mitochondria-directed targeting mechanism, is discussed in the context of current and previous investigations.

## 2. Results

### 2.1. BG95 Exerts Strong Anti-HCMV Activity In Vitro in the Absence of Cytotoxicity

**Figure 1:**
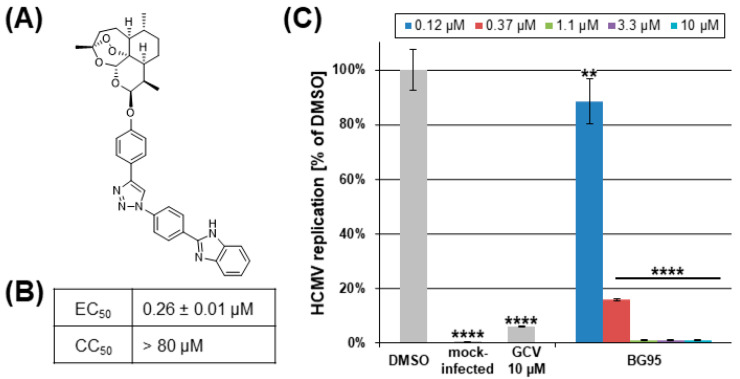
Assessment of BG95 anti-HCMV activity and cytotoxicity levels in vitro. (**A**) Chemical structure of BG95. (**B**) HCMV-specific EC_50_ and CC_50_ values of BG95 (the latter measured by neutral red assay; Figure S1B). (**C**) Anti-HCMV efficacy of BG95 measured by the HCMV GFP-based replication assay performed with primary human fibroblasts (HFFs), as compared to solvent control (DMSO), mock-infected cells and reference inhibitor ganciclovir (GCV, 10 µM). Statistical analysis was performed using ordinary One-way ANOVA and post-hoc Tukey’s test compared to DMSO. ** *p* < 0.01; **** *p* < 0.0001.

Hybrid compound BG95 represents a newly synthesized monomeric derivative of artemisinin (chemical class sesquiterpenes/trioxanes) possessing autofluorescence properties (Figure 1A). HCMV replication assays in primary human fibroblasts (HFFs) revealed an EC_50_ value in the submicromolar range (0.26 ± 0.01 µM; Figure 1B,C) in the absence of cytotoxicity (Figure S1; Figure 1B), indicating a strong and specific anti-HCMV efficacy. Short-term cytotoxicity at 1 d was assessed using the LDH release assay, demonstrating less than 15% LDH cytotoxicity signal for 100 µM of BG95, relative to the cytotoxicity-inducing positive control (0.1 µM staurosporine, STP), and a lack of measurable BG95 cytotoxicity at lower concentrations of 30 µM and 10 µM (Figure S1A). Long-term cytotoxicity at 3 d or 7 d was measured using the neutral red uptake assay, confirming a virtual absence of cytotoxicity for BG95 concentrations between 2.5 µM and 80 µM (Figure S1B).

### 2.2. BG95 Displays Pronounced Autofluorescence and Accumulates in Mitochondria

**Figure 2:**
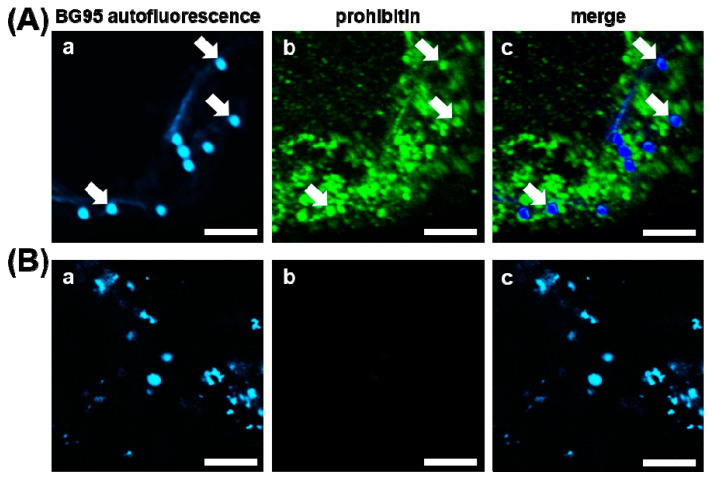
BG95 exhibits pronounced autofluorescence and localizes to mitochondria. (**A**) Autofluorescent BG95 bodies (image a,) were detectable using distinct settings of excitation at 405 nm and emission at 450–590 nm and colocalized with mitochondria of HCMV-infected HFFs stained via mitochondrial marker protein prohibitin (image b). White arrows indicate BG95 bodies and corresponding positions in other channels. (**B**) BG95 autofluorescence did not produce background in the excitation/emission settings employed for prohibitin staining (image b). Scale bars represent 5 µm.

Preliminary analysis of BG95 suggested autofluorescence of this compound with excitation and emission spectra similar to DAPI/Hoechst dye settings (excitation 405 nm, emission 450–590 nm). Autofluorescent accumulations, termed BG95 bodies (Figure 2A, image a, white arrows), could be consistently detected located in the cytoplasm of cells, as shown here in HCMV-infected HFFs at 3 d post-infection (p.i.). This autofluorescence could not be detected in any other channel (particularly not in the GFP or prohibitin settings at excitation/emission of 395/509 nm or 488/500–600 nm, respectively). This allowed for the simultaneous immunostaining of BG95-treated cells and thus enabled tracking analyses (Figure 2B). Using the mitochondrial marker protein prohibitin, complete colocalization of BG95 bodies with mitochondria was detected (Figure 2A, white arrows). In addition to serving as a mitochondrial marker, prohibitin also exerts important regulatory functions. Specifically, this multifunctional protein can act as a chaperone stabilizing mitochondrial proteins [62] as well as supporting respiratory supercomplex formation [63]. To further characterize the interaction of BG95 with mitochondria in the context of HCMV-infected HFFs, parameters of mitochondrial architecture, integrity, and membrane potential were assessed by various procedures specified below. 

### 2.3. Treatment with BG95 Induces Changes in Mitochondrial Structure, Which Are Distinct from Those Induced by HCMV Infection

**Figure 3:**
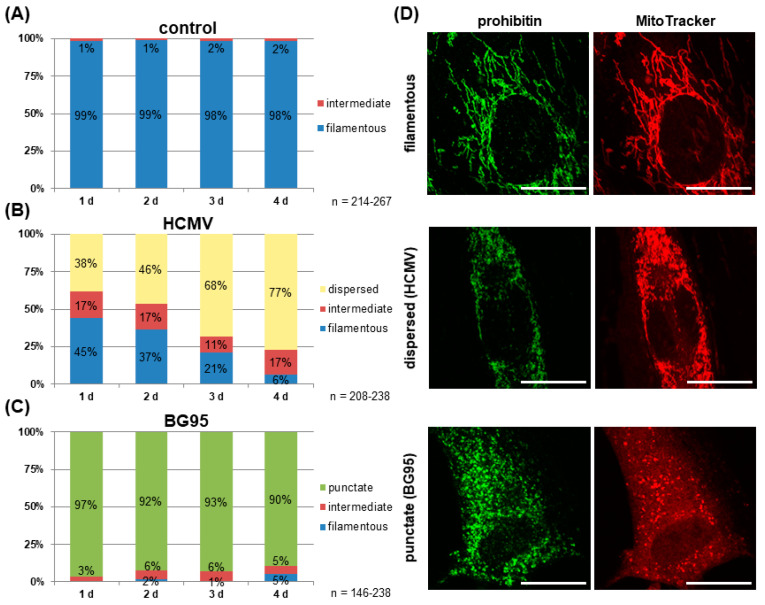
BG95 treatment and HCMV infection induce distinct changes in mitochondrial morphology, i.e., punctate or dispersed patterns, respectively. The mitochondrial architecture was assessed using confocal microscopy and both prohibitin and MitoTracker staining and was classified into four categories based on visual monitoring and cell counting, i.e., filamentous/normal, dispersed (HCMV), punctate (BG95) and intermediate. Cells were fixed and examined at four consecutive days after infection with the beginning of treatment as indicated. (**A**) Mitochondria of mock-infected, untreated HFFs showed a filamentous structure, which did not change over time. (**B**) Infection with HCMV AD169 led to mitochondrial fragmentation towards a dispersed phenotype, which increased over time in correlation to virus production and spread. (**C**) BG95 treatment of HFFs (10 µM) induced an invariable punctate architecture in mitochondria in the vast majority of cells. (**D**) Representative pictures illustrating the changes of mitochondrial morphology quantitated in A–C. Scale bars represent 20 µm.

Mitochondria of cultured HFFs show a filamentous, thread-like architecture [64,65]. In this study, this characteristic mitochondrial structure could be visualized by both anti-prohibitin antibody staining and MitoTracker dye (Figure 3D). Optical evaluation of >140 cells per day at four consecutive days confirmed the unaltered filamentous phenotype of mitochondrial architecture in control cells (Figure 3A). Infection with HCMV (MOI 1.0–2.0), in comparison, resulted in a disintegration of these filamentous mitochondrial structures in approx. 40% of cells at 1 d p.i., towards a dispersed morphology (Figure 3B,D). A temporal increase in the percentage of cells displaying this dispersed phenotype could be detected, with 77% of cells exhibiting this HCMV-induced alteration at 4 d p.i. Control fluorescence stainings with the antibody against viral immediate-early protein 1 (IE1) confirmed a nearly complete productive infection of the cell layer at 4 d p.i. (Figure S2). In contrast to this HCMV-specific mitochondrial phenotype, a drug-induced morphological change was observed in mock-infected HFFs treated with 10 μM of BG95. Here, the vast majority of cells (>95%) displayed small, broken-up mitochondria of a punctate phenotype at 1 d p.i. (Figure 3C,D). No substantial change was observed over time, although a slight increase in the percentage of filamentous and intermediate morphology was detected at 4 d p.i. Importantly, an identical phenotype of mitochondrial architecture was observed in HFFs treated with the parental compound ART and additional ART-like derivatives (Figure S3). 

### 2.4. BG95 Induces Mitochondrial Changes but Does Not Induce Apoptosis

**Figure 4:**
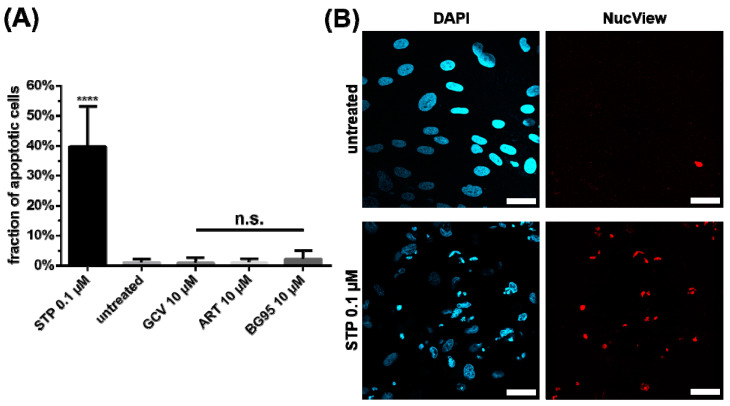
BG95 and related trioxane compounds do not induce apoptosis in primary human fibroblasts. (**A**) HFFs were treated with the indicated concentrations of compounds or remained untreated for 3 d, after which they were subjected to fixation, NucView, and DAPI staining. The fraction of NucView-positive cells were counted in at least 5 images per compound and data are given as mean + SD. Staurosporine (STP, 0.1 µM) was used as a positive control. Statistical analysis was performed using ordinary One-way ANOVA followed by post-hoc Tukey’s test compared to untreated. **** *p* < 0.0001; n.s., not significant. (**B**) Representative images of DAPI and NucView channels comparing untreated versus STP-treated cells. Scale bars represent 20 µm.

Notably, the BG95-induced morphological change in mitochondria was not associated with mitochondria-triggered apoptotic cell death. A quantitative assessment of apoptosis induction in HFFs, MEFs and HeLa cells was conducted using the NucView staining technique, which labels apoptotic cells dependent on active caspase 3. While treatment with STP, a known potent inducer of the apoptotic pathway, elicited a positive signal in 40% of HFFs, neither BG95 nor the parental compound ART showed a similar signal of apoptosis (Figure 4; untreated and GCV-treated cells were used as controls). Likewise, very low levels of apoptosis were measured for ART and BG95 in MEFs and HeLa cells (Figure S4). In all cell types, the fraction of apoptotic cells induced by BG95 remained at a marginal level below 5%, indicating that BG95 and related trioxane compounds are free of apoptotic or cytotoxic effects.

### 2.5. BG95 Localizes to Mitochondria and Leads to a Loss of Physiological Membrane Potential

**Figure 5:**
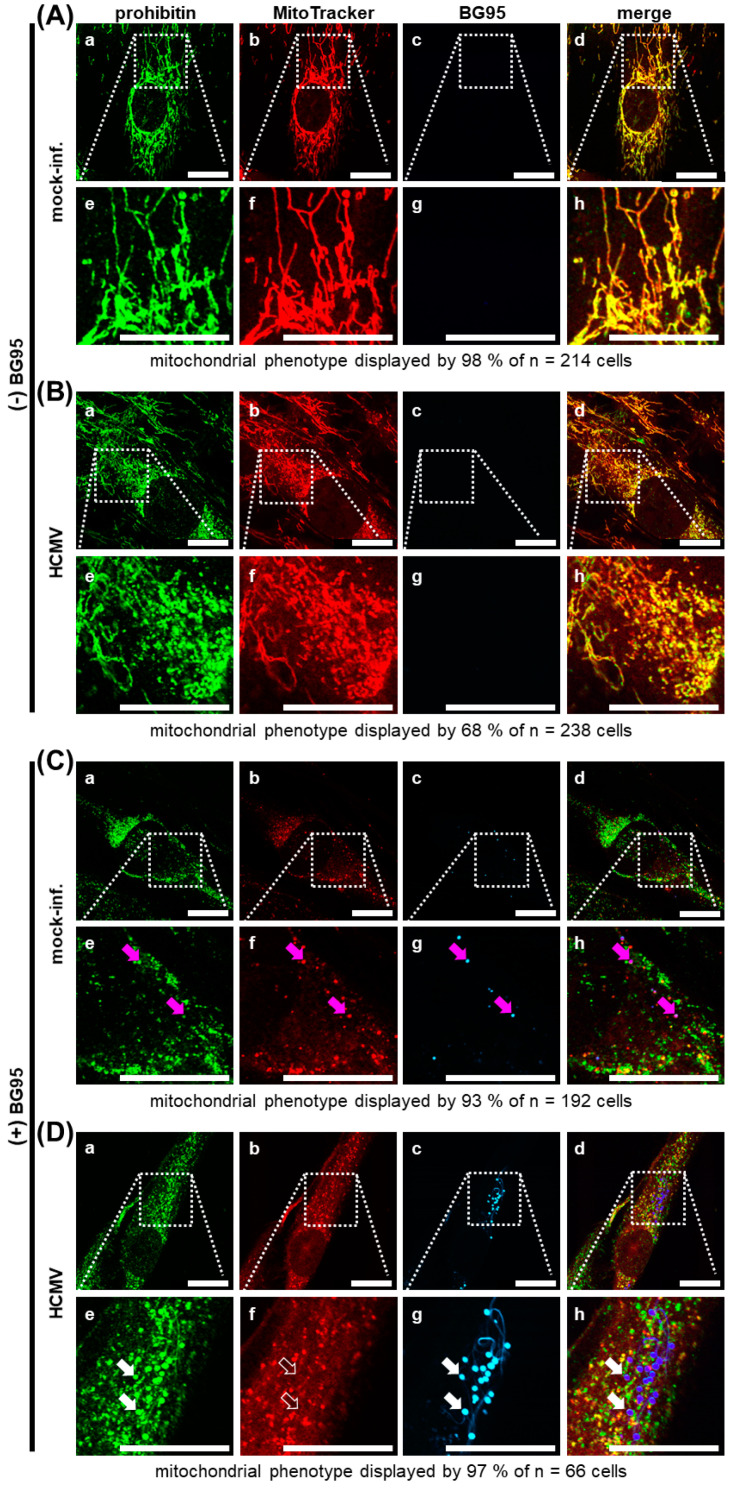
Autofluorescent BG95 accumulates in characteristic intramitochondrial bodies converting mitochondria to an inactive state. (**A**) Mitochondria of untreated, mock-infected cells consistently display a filamentous structure as visualized by overlapping prohibitin and MitoTracker signals (compare also Figure 3). (**B**) HCMV infection induces dispersed mitochondria as visualized by overlapping prohibitin and MitoTracker signals (see drug autofluorescence-negative images c and g as a control). (**C**) Treatment with 10 µM BG95 causes a punctate mitochondrial phenotype and drug autofluorescence. Magenta arrows indicate strong BG95 autofluorescence with the corresponding positions in other channels. Note some loss of overlap between prohibitin and MitoTracker signals. (**D**) Combined, BG95 treatment and HCMV infection led to strongly autofluorescent BG95 bodies colocalized with prohibitin, but not with the MitoTracker signal. White arrows indicate exemplary BG95 bodies, which display a lack of MitoTracker signal at these specific sites (unfilled arrows). White dotted frames indicate the area enlarged in the respective inset, connected by dotted lines. Scale bars represent 20 µm.

To further characterize the impact of BG95 treatment, two distinct approaches to visualize mitochondria were employed, immunostaining of the marker protein prohibitin as well as MitoTracker dye staining, which permeates mitochondria depending on intact membrane potential. Control panels of HFFs, i.e., mock-infected and drug-untreated cells, displayed filamentous mitochondria as described before (Figure 5A), with no autofluorescence signal detectable in the channel designated BG95 (Figure 5A, images c and g). Strong merge signals obtained for prohibitin and MitoTracker confirmed the applicability of the dual staining procedure (Figure 5A, images d and h). HCMV-infected, drug-untreated cells showed virus-induced mitochondrial fragmentation towards a dispersed phenotype in most cells (Figure 5B, compare also Figure 3D), with only some cells retaining their physiological filamentous structure. In mock-infected cells, treated with 10 µM of BG95, drug-specific autofluorescence was detected (Figure 5C, images c and g), colocalizing with both prohibitin and MitoTracker signals (Figure 5C, magenta arrows), underlining our hypothesis of mitochondrial targeting of BG95. The BG95-induced punctate mitochondrial phenotype could be observed here in both the prohibitin and MitoTracker staining. Importantly, the distinct overlap of these two staining signals was found reduced in a portion of mitochondria, which remained prohibitin positive, but showed no MitoTracker signal (Figure 5C, images e, f, and h). This lack of MitoTracker was most pronounced in HCMV-infected, BG95-treated HFFs (Figure 5D). In this setting, strong autofluorescent BG95 bodies of uniform size and shape were observed in the majority of cells, partly accompanied by yet unidentified autofluorescent thread-like structures (Figure 5D, images c and g). These BG95 accumulations strongly colocalized with prohibitin signal (Figure 5D, filled white arrows), as well as with an additional mitochondrial marker protein, ATP5A1 (Figure S5, white arrows), but did not colocalize with MitoTracker signal (Figure 5D, unfilled white arrows), indicating a change in membrane potential and mitochondrial electron transport chain (ETC) activity. As an independent control experiment, human glioblastoma-astrocytoma U373 cells were likewise BG95-treated and analyzed. The appearance of BG95 bodies in these cells, colocalized with the mitochondrial marker prohibitin closely mirrored the results in HFFs, with no visible cell-specific differences (Table S1). Thus, data strongly suggest that the intramitochondrial accumulation of BG95 induces a loss of ETC activity, especially in HCMV-infected cells.

**Figure 6:**
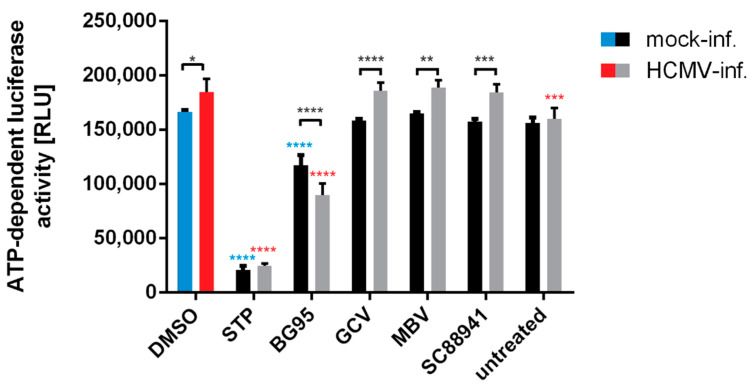
Treatment with BG95, but not control drugs, induces a significant reduction of ATP levels in both mock-infected and HCMV-infected cells, while HCMV infection raises ATP levels. HFFs were infected with HCMV AD169 at an MOI of 0.5 or remained mock-infected. Cells were treated with BG95, reference drug GCV (both 10 µM), two investigational antivirals effective at low doses (MBV and SC88941, both 3 µM), the apoptosis-inducing compound STP (0.1 µM) or DMSO as solvent control. ATP-dependent luciferase activity was quantitated in quadruplicates at 3 d p.i. using the CellTiter-Glo^®^ Luminescent Cell Viability Assay. Statistical analysis was performed using ordinary Two-way ANOVA and post-hoc Tukey’s test (blue asterisks represent the significance of mock-infected compared to mock-infected DMSO control, red asterisks represent the significance of HCMV-infected compared to HCMV-infected DMSO control; black asterisks represent the significance of mock-infected versus HCMV-infected pairs of samples). ** *p* < 0.01; *** *p* < 0.001; **** *p* < 0.0001; RLU, relative light units.

To further illustrate the targeting of mitochondrial activity and integrity, a specific measurement of intracellular ATP levels under drug treatment was performed. To this end, an established test system based on the ATP-dependent luciferase reporter activity (CellTiter-Glo^®^ Luminescent Cell Viability Assay; Promega, Madison, WI, USA) was applied using HCMV-infected and mock-infected HFFs. In both cases, cells were subjected to BG95 treatment as compared to reference drugs including GCV, a DMSO solvent control, and an untreated control (Figure 6). Data indicate that HCMV infection in general has a stimulatory impact on ATP levels in HFFs. Importantly, BG95 showed an inhibitory effect on ATP-dependent reporter signals, which was distinct from the treatment with reference drugs, such as GCV, maribavir (MBV, an inhibitor of viral kinase activity; [66]) or SC88941 (a host-directed antiviral compound possessing a complex spectrum of target protein binding; [67]). Thus, the ATP-reducing activity of BG95 was found specific compared to other antiviral drugs and was similarly detectable for HCMV-infected and mock-infected cells. This finding supports our concept that BG95 targets mitochondria and exerts an inhibitory effect on mitochondrial ATP production. Combined, the data underline the mitochondrial targeting of BG95 and related trioxane compounds, including a regulatory impact on the mitochondrial activity as shown by compound-induced alteration of ATP levels.

### 2.6. The Morphological Conversion of Mitochondria Induced by BG95 or Related Trioxanes Is Dependent on Drug Concentration and the Presence of an Intramolecular Endoperoxide Bridge

**Figure 7:**
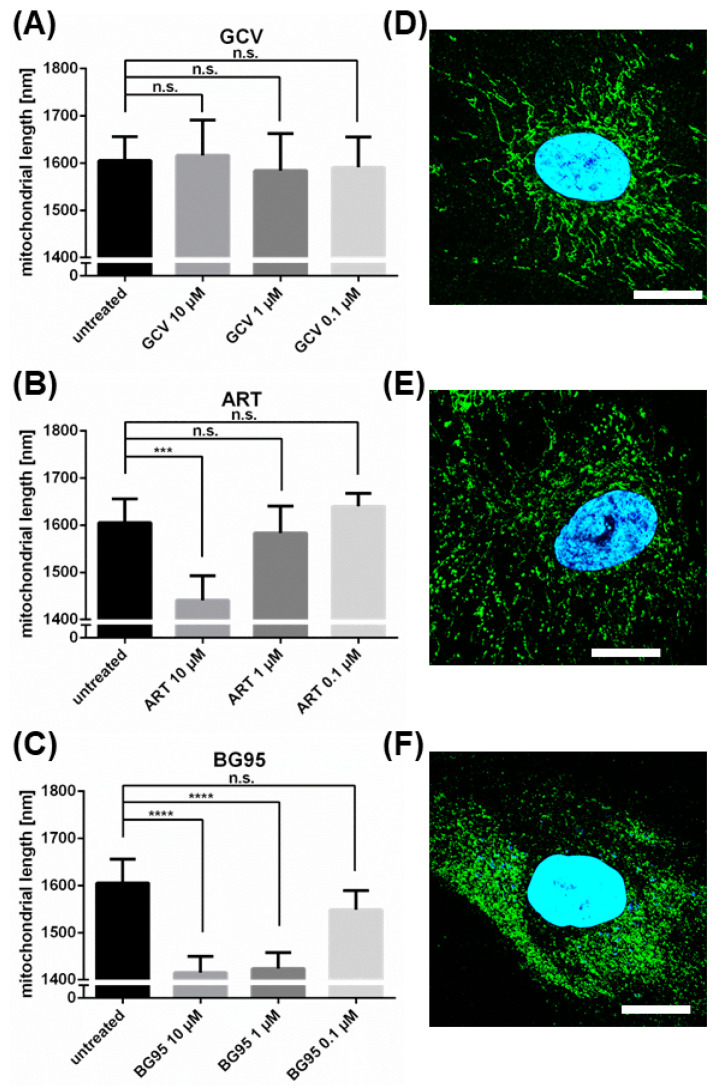
ART derivatives, but not control drug GCV, induce significant dose-dependent shortening of mitochondria in primary human fibroblasts. HFFs were treated with the indicated concentrations of compounds or remained untreated for 3 d, after which they were fixed and stained using DAPI and antibody against mitochondrial marker protein prohibitin. (**A**) Treatment with control drug GCV did not change mitochondrial architecture, as shown by no significant alteration to mean mitochondrial length. (**B**) Treatment with 10 µM of ART resulted in a statistically significant shortening of mitochondria. (**C**) Treatment with 10 µM as well as 1 µM of BG95 led to a highly significant shortening of mean mitochondrial length. The length of mitochondria was assessed using the FilamentDetector plugin of ImageJ. Mean mitochondrial length was measured in at least 5 individual images and >100 cells per compound/concentration. Data are given as the mean of mitochondrial length + SD. Statistical analysis was performed using ordinary One-way ANOVA followed by post-hoc Tukey’s test compared to untreated. **** *p* < 0.0001; *** *p* < 0.001; n.s., not significant (**D**–**F**) Representative confocal images of mitochondrial structure (green) in HFFs treated with GCV, ART or BG95, respectively (all 10 µM). Note in (**F**) the BG95 autofluorescence (blue) signal colocalized with mitochondria (green).

A quantitative assessment of BG95-/related trioxane-induced effects on mitochondrial morphology was conducted by using the FilamentDetector plugin of ImageJ software. As described above, a complete conversion of filamentous mitochondria towards a punctate morphology was observed following the drug treatment of HFFs (in the absence of HCMV to exclude infection-dependent effects). The mitochondrial length in five panels with at least 100 cells between them was assessed per treatment and is presented as mean value + SD (Figure 7). Importantly, both trioxane compounds analyzed in this setting, BG95 and ART at 10 µM, showed statistically significant activity of mitochondrial filament conversion compared to untreated control cells (Figure 7B,C). This conversion was not observed for the reference compound GCV (Figure 7A). Notably, BG95 showed high efficacy even at a low concentration of 1 µM, whereas ART activity was only moderate and not detectable at 1 µM. This difference in mitochondrial conversion correlates with the different anti-HCMV efficacies of ART (EC_50_ = 5.41 µM [27]) and BG95 (EC_50_ = 0.26 µM, Figure 1B,C). The concentration of 1 µM of ART is thus inactive in changing mitochondrial architecture and has been shown not to inhibit HCMV replication [27]. In contrast, 1 µM of BG95 significantly altered mitochondrial architecture and also proved to be effective in inhibiting HCMV replication (Figure 1C). The quantitative evaluation of mitochondrial architecture also perfectly correlated with the qualitative patterns observed visually in independent confocal images (Figure 7D–F). Another trioxane analog chemically closely related to BG95, BG90, was additionally analyzed (BG90 synthesis and chemical structure will be published elsewhere; Herrmann et al., manuscript in preparation). Similar to BG95 and ART, BG90 also induced a significant conversion of mitochondrial filaments to punctate structures at concentrations between 1–10 µM.

**Figure 8:**
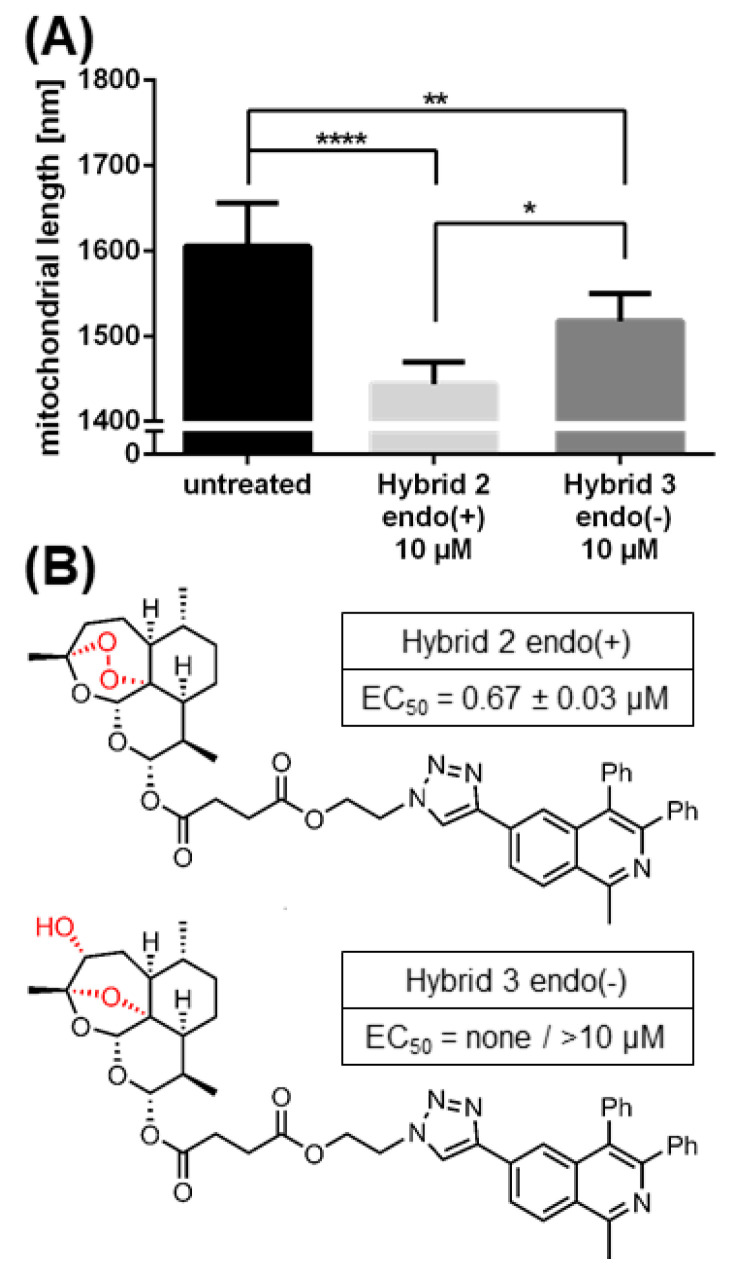
Conversion of mitochondrial architecture by BG95-related trioxane compounds is largely dependent on their endoperoxide bridge. HFFs were treated with the indicated concentrations of compounds or remained untreated for 3 d, after which they were fixed and stained. Staining procedure and assessment of mean mitochondrial length was performed as described for Figure 5. (**A**) Both Hybrid 2 endo(+) and Hybrid 3 endo(−) led to a decrease in mean mitochondrial length, with the endoperoxide bridge-intact Hybrid 2 changing mitochondria to a significantly higher degree than the endoperoxide bridge-negative Hybrid 3. Mean mitochondrial length was measured in at least 5 individual pictures and >100 cells per compound. Data are given as the mean of mitochondrial length + SD. Statistical analysis was performed using ordinary One-way ANOVA followed by post-hoc Tukey’s test compared to untreated. **** *p* < 0.0001; ** *p* < 0.01; * *p* < 0.05 (**B**) Chemical structures and anti-HCMV efficacy of ART derivatives Hybrid 2 endo(+) and Hybrid 3 endo(−), with the presence or absence of an endoperoxide bridge highlighted in red.

To address the role of the active determinant of trioxane/sesquiterpene compounds, such as their common endoperoxide bridge, a comparison of two related compounds, differing in this specific determinant [68,69], was performed in parallel (Figure 8). Importantly, Hybrid 2 endo(+) showed a highly potent conversion of mitochondrial filaments, while the activity of Hybrid 3 endo(−) was substantially lower and remained at intermediate levels (Figure 8A). The finding paralleled the anti-HCMV activity of the two compounds, which was strong for Hybrid 2 endo(+), with an EC_50_ value of 0.67 ± 0.03 µM, but was non-existent for Hybrid 3 endo(−), as analyzed up to 10 µM (Figure 8B). This correlation strongly supported our concept that the endoperoxide bridge of trioxane compounds is important for antiviral activity. Interestingly, compound Hybrid 3 endo(−) was not completely inactive in mitochondrial conversion activity, despite its lack of antiviral activity. This may indicate that the antiviral activity, which proved to be based on the drug binding to multiple target proteins [61], is mostly linked to an active endoperoxide bridge. In contrast, the activity of mitochondrial conversion is only partly endoperoxide bridge-dependent and appears to include an additional endoperoxide bridge-independent target binding property. This seems plausible, as the covalent protein binding properties of trioxane compounds can principally be conferred through the endoperoxide bridge. In addition, however, the compounds’ bioactivity may be mediated by a noncovalent mode of binding to other proteins, possibly mediated through other compound moieties [61,70,71,72,73]. Thus, the strong in vitro efficacy and concentration-dependent antiviral activity of this class of compounds correlates at least in part with the intramolecular endoperoxide bridge. Combined, several lines of evidence point to a causative link between BG95 antiviral activity, its property of accumulation in mitochondria associated with a putative inhibitory effect on mitochondrial membrane potential, and an active endoperoxide bridge.

## 3. Discussion

Mitochondria play an essential role in various cellular processes such as the production of biosynthetic precursors, signaling pathways, or regulation of intrinsic apoptosis. The most prominent function is the supply of chemical energy in the form of adenosine triphosphate (ATP) by oxidative phosphorylation. As cellular energy production is of crucial importance for viral replication, several viruses have developed strategies to target mitochondria and either induce or inhibit mitochondrial functions [74]. HCMV infection has been shown to increase mitochondrial DNA synthesis [75] and lipid turnover [76], indicating a stimulating effect. Specifically, HCMV replication requires increased ATP levels, and the upregulation of genes responsible for oxidative phosphorylation on transcriptional and translational levels has been discussed in this context [77,78,79]. It has been postulated that an enhanced mitochondrial energy production might be a driving force for increased ATP levels, but surprisingly, this could not be fully confirmed by currently available assays [80,81,82,83]. Alternatively, increased energy production may result from virus-induced glycolysis supplying the required ATP. Our current experimentation based on the measurement of intracellular ATP levels under drug treatment further illustrated this point as described above (Figure 6), detecting consistently raised ATP levels in HCMV-infected versus uninfected cells. Furthermore, endoplasmic reticulum-mitochondrial contact sites increase in number upon HCMV infection [84], which is another indicator pointing to the virus-induced enhancement of mitochondrial functionality [85]. To assess HCMV- and drug-induced regulatory impacts on mitochondria, two distinct methods of visualization were employed in this study. Firstly, the mitochondrial protein prohibitin was stained as a marker of intracellular drug localization. Secondly, the MitoTracker Deep Red fluorescent dye was used, which accumulates in mitochondria depending on an intact membrane potential. As a response to HCMV infection, 38% of HFFs displayed fragmented, dispersed mitochondria, which stood in contrast to the filamentous phenotype exhibited by uninfected cells. This fragmentation of the mitochondrial network by HCMV has been observed before and is probably caused by the viral anti-apoptotic protein pUL37, which is actively transported into mitochondria [86,87,88]. Accordingly, the percentage of cells displaying these fragmented mitochondria increased to 46%, 68%, and 77% at 2, 3, and 4 d p.i., respectively. This morphological alteration reflected the continuous remodeling of cellular structures by viral replication and the spread of infection in the fibroblast monolayer. It should be emphasized that the findings on drug-induced mitochondrial changes may directly imply potential induction of apoptosis and cytotoxicity. This constitutes a highly relevant question, particularly in view of significant toxicity and applicability limitations of many, if not all, current HCMV-specific therapeutics. Notably, the presented assessment of apoptotic signals in cultured cells clearly demonstrated a lack of such potential drug side-effects for BG95 and related trioxanes. Serial microscopic inspections, including trypan blue and neutral red staining procedures excluded the possibility of substantial induction of cytotoxicity, too [17,27,68,89]. In fact, trioxane parental drugs like artemisinin, artesunate and artemether are widely used for therapies exhibiting mostly low level-toxicity, as shown by meta-analysis. Their continued use in anti-infective applications, particularly treating malaria disease, has proven to be safe and of substantial clinical benefit [17,90,91]. 

The mitochondrial structure was not only altered by HCMV infection, but also by treatment with compounds of the trioxane/sesquiterpene class including BG95. In contrast to the dispersed phenotype induced by HCMV, however, cells exhibited small and round mitochondria of a punctate phenotype. Importantly, the change induced by 10 µM of BG95 was present in nearly all cells and already one day after treatment. Contrasting the virus-induced fragmentation, this compound-associated conversion was consistently observed over time (97%, 92%, 93%, 90% at 1, 2, 3, and 4 d of treatment, respectively). A minute decrease in the percentage of the drug-affected cells was detectable, which corresponded with the reconstitution of filamentous mitochondria (0%, 2%, 1%, 5% at 1, 2, 3, and 4 d of treatment, respectively). This phenomenon may be a consequence of the intracellular turnover of BG95 to a less effective metabolite, consequently leading to some minor reconstitution of the physiological mitochondria structure. 

Due to its pronounced autofluorescence properties, BG95 could be tracked and assessed in its intracellular localization via confocal microscopy. The compound accumulated in characteristic aggregates of uniform size and shape termed BG95 bodies. Importantly, these BG95 bodies were found to colocalize with a strong signal of the mitochondrial marker protein prohibitin, indicating mitochondrial localization. Pronounced effects of artesunate-like compounds on mitochondria have been demonstrated in previous reports [92,93,94], which are consistent and complementary with the microscopic investigations of the present study. Interestingly, earlier reports stressed that knock-out of prohibitin led to fragmented mitochondria [95]. The identified colocalization of BG95 bodies with the prohibitin signal suggests a correlation between these findings and the possible interaction of BG95 with prohibitin. Preliminary experiments in our hands, aiming at the identification of cellular effectors of BG95-induced mitochondrial alteration, indicated a BG95-induced modification of cytochrome C towards a slower-migrating band pattern in the denaturing SDS-PAGE/Western blot procedure [96]. Posttranslational modifications (PTMs) provide a plausible explanation for such alterations in migration behaviour. PTMs of cytochrome C have been described before [97] and the present migration pattern suggests the induction of phosphorylated cytochrome C. Investigations have shown that cytochrome C phosphorylation can have a strong regulatory impact on the mitochondrial membrane physiology and may thus possibly represent part of the BG95-specific mechanism of action [97], which needs to be addressed by more detailed experimentation in the near future.

Based on our combined findings, we postulate a mitochondrial targeting of BG95 and related derivatives possessing pronounced antiviral efficacies both in vitro and in vivo [20,49,51,61,68,98,99]. 

Intriguingly, mitochondria that showed strong BG95 signals were consistently found to be devoid of MitoTracker signal. As the uptake of MitoTracker dye is dependent on membrane potential, this can be interpreted as a loss of integrity and activity in these mitochondria, prompting our hypothesis that BG95 inhibits mitochondrial function. As mentioned before, decreased mitochondrial membrane potential can initiate apoptosis [100], however, no induction of apoptosis by BG95, nor overall cytotoxic effects were measurable in our experiments.

Importantly, a decrease in mitochondrial membrane potential due to the parental compound artesunate has already been demonstrated in plasmodium parasites [101]. As HCMV relies on the upregulation of mitochondrial activity to provide sufficient levels of metabolic activity and energy for viral replication and progeny production, a loss in mitochondrial membrane potential should significantly interfere with the efficiency of viral replication. This indicates mitochondrial targeting as a general mechanistic hallmark of this class of compounds. In terms of antiviral activity, this finding further illustrates our concept of the multifactorial mechanism of these drugs, including a mitochondria-directed mode of target protein inhibition, in addition to the described signaling/NF-ĸB effects, the binding to exportins and other potential targets [61]. Thus, the present study clarifies the previously poorly characterized impact of BG95 and related compounds on mitochondrial structure and their state of activity in human cells, strongly suggesting a refined concept of antiviral mode of action.

## 4. Materials and Methods

### 4.1. Antibodies

The following antibodies were used in this study: monoclonal rabbit antibody against prohibitin (EP2804Y; Abcam, Cambridge, UK), monoclonal mouse antibodies against ATP5A1 (7H10BD4F9; Thermo Fisher Scientific, Waltham, MA, USA), cytochrome C (IgG2b SC 13156; SantaCruz, Dallas, TX, USA) and β–actin (AC-15, Merck, Darmstadt, Germany). Goat anti-rabbit Alexa 488-conjugated antibody (A-11034, Thermo Fisher Scientific, Waltham, MA, USA) was used as a secondary antibody for indirect immunofluorescence analysis. 

### 4.2. Antiviral Compounds

Artemisinin/artesunate derivatives, including BG95, were synthesized in the laboratory of S.B.T., dissolved in DMSO (Merck, Darmstadt, Germany) and stored at −20 °C.

### 4.3. Cells and Viruses

Primary human foreskin fibroblasts (HFFs, derived from clinical samples, Children’s Hospital, Erlangen, Germany) were grown in Eagle’s Minimal Essential Medium (MEM) supplemented with 350 μg/mL glutamine (both Thermo Fisher Scientific, Waltham, MA, USA), 10% (*v*/*v*) fetal calf serum (FCS, Capricorn, Ebsdorfergrund, Germany) and 10 μg/mL gentamycin. Human U373 glioblastoma-astrocytoma cells were cultivated in Dulbecco’s modified Eagle’s medium (DMEM), supplemented with 350 μg/mL glutamine, 10% (*v*/*v*) FCS and 10 μg/mL gentamicin. Cultured cells were maintained at 37 °C, 5% CO_2_, and 80% humidity. HCMV strain AD169 variant UK [102] was propagated in HFFs and employed for immunofluorescence, while recombinant HCMV AD169 expressing green fluorescent protein (AD169-GFP [103]) was used for in vitro replication assays.

### 4.4. HCMV GFP-Based Replication Assay

Inhibition of viral replication was assayed by the use of recombinant HCMV expressing green fluorescent protein (GFP) as described previously [27,103,104]. In brief, HFFs were cultivated in 12-well plates (2 × 10^5^ cells per well) and infected with HCMV AD169-GFP at an MOI of 0.25 (i.e., 25% GFP-forming dose of a multi-round infection measured at 7 d p.i.). After virus adsorption of 90 min, the inoculum was replaced by medium supplemented with BG95. 7 d post-infection (p.i.) cells were lysed by the addition of 200 µL lysis buffer. Subsequently, cell suspensions were mixed and transferred to a 96-well plate. Centrifugation was performed at 3000 rpm for 15 min and clear lysates were subjected to automated GFP quantitation in a Victor X4 microplate reader (PerkinElmer, Waltham, MA, USA). All infections were performed in duplicate, GFP quantitations were performed in quadruplicate and antiviral efficacy is expressed as mean EC_50_ values ± SD.

### 4.5. Assessment of Cell Viability

Acute short-term cytotoxicity (1 d) was assessed by using the standard lactate dehydrogenase (LDH) release assay as described earlier [105]. Long-term cytotoxicity was assessed by the measurement of neutral red uptake [106]. HFFs were treated with BG95 at increasing concentrations for 3 d or 7 d. Cells were incubated with 40 μg/mL neutral red solution (Merck, Darmstadt, Germany) for 3 h at 37 °C. Cells were destained with 1% acetic acid in 50% ethanol solution before neutral red signals were quantitated by photometric measurement (excitation/emission at 560/630 nm) in a Victor X4 microplate reader (PerkinElmer, Waltham, MA, USA). Assays were performed in triplicate and cytotoxicity levels are expressed as mean CC_50_ values ± SD. The induction of apoptosis was assessed by using the commercially available BioTracker NucView^®^ 530 Red Caspase-3 Dye staining kit (Merck, Darmstadt, Germany) according to the instructions by the manufacturer. In brief, approximately 2 × 10^5^ cells per well were grown on coverslips in 6-well culture plates and treated with compound diluted in media beginning 1 d after seeding. At 3 d after the onset of treatment, the medium was removed and cells incubated with 5 µM NucView reagent in medium (40 min, 37 °C); subsequently, cells were fixed and stained as described in 4.6.

### 4.6. Indirect Immunofluorescence Assay Using MitoTracker Staining and Laser Scanning Microscopy

For immunofluorescence detection, approx. 2 × 10^5^ HFFs per well were grown on coverslips in 6-well culture plates and used for infection with HCMV strain AD169 at an MOI of 1.0–2.0 or remained mock-infected. BG95 (10 µM) was added immediately after infection. At the time points p.i. indicated, cells were incubated with 150 nM MitoTracker™ Deep Red FM dye (Thermo Fisher Scientific, Waltham, MA, USA) in standard culture medium (40 min, 37 °C), fixed with 4% paraformaldehyde solution (10 min, room temperature) and permeabilized by incubation with 0.2% Triton X-100 solution (20 min, 4 °C). The concentration of 10 µM of BG95 was chosen so that drug-specific fluorescence could be visualized in the absence of cytotoxicity (at lower concentrations near the EC_50_ value, drug-specific fluorescence could not be detected). Nonspecific staining was blocked by incubation with 2 mg/mL human γ-globulin (Cohn fraction II, Merck, Darmstadt, Germany; 30 min, 37 °C). Indirect immunofluorescence staining was performed by incubation with primary antibody diluted in PBS (60 min, 37 °C), washing, and subsequent incubation with diluted dye-conjugated secondary antibody (30 min, 37 °C). After further washing steps, cells were mounted with VECTASHIELD^®^ Mounting Medium with/without DAPI (Vector Laboratories, Burlingame, CA, USA), before glass coverslips were sealed using nail polish. Confocal laser-scanning microscopy was performed with a TCS SP5 microscope using a 63× HCX PL APO CS oil immersion objective lens (Leica Microsystems, Mannheim, Germany). Images were processed using the LAS AF software (version 2.6.0 build 7266; Leica Microsystems, Mannheim, Germany) and edited with Photoshop CS5 (Adobe, San José, CA, USA).

### 4.7. Quantitation of Drug-Induced Morphological Changes of Mitochondria by Using the Filamentdetector Plugin of ImageJ Software

To quantitate changes of mitochondrial architecture in HFFs, approx. 2 × 10^5^ HFFs per well were grown on coverslips in 6-well culture plates. Indicated compounds (10 µM, 1 µM or 0.1 µM) were added 1 d after seeding, or cells were left untreated. At 3 d after the onset of treatment, cells were fixed and stained with anti-prohibitin antibody and DAPI for the use in confocal imaging as described in 4.6. Pictures were edited using Photoshop CS5 and ImageJ, applying identical edits to all images. Subsequently, the mean length of mitochondria in one image (using prohibitin signal as a correlate) was measured via the FilamentDetector plugin (version 0.4.8) of ImageJ software (v1.52p) using standard settings, except for a Sigma value = 3. Detected filaments were exported and filtered, removing filaments with a sinuosity value ≥1.2 to exclude circular filaments falsely detected in mitochondrial aggregates, as well as filaments with a length ≤1000 nm to exclude falsely detected filaments in microscopic artefacts.

### 4.8. Measurement of Intracellular ATP Levels

HFFs were seeded in 96-well culture plates at 1.35 × 10^4^ cells/well and infected one day later with HCMV-AD169 at an MOI of 0.5 or remained mock-infected. Indicated compounds (10 µM, 3 µM or 0.1 µM) were added immediately after infection, or cells remained untreated. Levels of ATP were quantitated in quadruplicates at 3 d p.i. in a Victor X4 microplate reader using the CellTiter-Glo^®^ Luminescent Cell Viability Assay (Promega, Madison, WI, USA) according to manufacturer’s protocol.

### 4.9. Analysis of Mitochondrial Proteins Using Differential Centrifugation and SDS-PAGE/Western Blot

HFFs were seeded in 6-well culture plates at 250,000 cells/well and infected one day later with HCMV-AD169 at an MOI of 2 or remained mock-infected. Beginning immediately after infection, cells were treated with the indicated compounds; solvent DMSO or 1 µM of staurosporine (STP) were used as controls. Cells were harvested 3 d p.i., sonicated in isolation buffer (0.25 M sucrose, 10 mM HEPES pH 7.5) and used for mitochondrial/nuclear enrichment [96]. Proteins were analyzed via SDS-PAGE and Western blot procedures using standard settings as described previously [107] with antibodies against the indicated antigens.

## 5. Conclusions

In this study we demonstrated the strong anticytomegaloviral efficacy of the new artemisinin-derived compound BG95 in the absence of apoptosis induction and cytotoxicity. Tracking analyses employing the autofluorescent properties of BG95 revealed mitochondrial accumulation, as well as a reduction of mitochondrial membrane potential in BG95-treated HCMV-infected primary human fibroblasts. Concentration-dependent and statistically significant alterations of mitochondrial architecture induced by BG95 and related trioxane compounds could be shown and partially linked to an intact intramolecular endoperoxide bridge. Combined, the present study underlines the important role of mitochondria in the multifaceted, host-directed antiviral mechanism of action of trioxane compounds.

## Supplementary Materials

Supplementary materials can be found at https://www.mdpi.com/1422-0067/21/15/5578/s1. Table S1. Characteristics of mitochondria under treatment with BG95/ART compounds in HFFs and U373 cells. Figure S1. BG95 shows no cytotoxic activity after incubation periods of 1, 3 and 7 d. Figure S2. Productive infection of HFFs with HCMV strain AD169 was visualized by the detection of IE1 expression. Figure S3. Artesunate and its derivatives TF27 and LH54 elicit changes to mitochondrial architecture of HFFs. Figure S4. BG95 does not induce apoptosis in primary human or murine fibroblasts, i.e., HFFs (A) or MEFs (B), and HeLa cells (C). Figure S5. Autofluorescent BG95 accumulates in characteristic intramitochondrial bodies as indicated by the ATP5A1 marker protein.

## Abbreviations

ART	Artesunate
ATP	adenosine triphosphate
CDV	cidofovir
d	day(s)
DAPI	4′,6-diamidino-2-phenylindole
DMEM	Dulbecco’s modified Eagle’s medium
DMSO	Dimethyl sulfoxide
DNA	deoxyribonucleic acid
ETC	electron transport chain
FCS	fetal calf serum
FOS	foscarnet
GCV	ganciclovir
GFP	green fluorescent protein
HCMV	human cytomegalovirus
HFF	human foreskin fibroblast
IE1	immediate-early protein 1
LDH	lactate dehydrogenase
MBV	maribavir
MEF	murine embryonic fibroblast
MEM	Eagle’s Minimal Essential medium
min	minute(s)
MOI	multiplicity of infection
p.i.	post-infection
rpm	rotations per minute
SD	standard deviation
STP	staurosporine
VGCV	valganciclovir
