# Supplementary material for: The Artemisinin-Derived Autofluorescent Compound BG95 Exerts Strong Anticytomegaloviral Activity Based on a Mitochondrial Targeting Mechanism
Source: Int J Mol Sci. 2020 Aug 4;21(15):5578. doi: 10.3390/ijms21155578 (PMC7432203; doi:10.3390/ijms21155578)
Supplement: Supplementary file 1 [file ijms-21-05578-s001.pdf]

International Journal of *Molecular Sciences*

Supplementary Materials

Article

The artemisinin-derived autofluorescent compound BG95 exerts anticytomegaloviral activity based on a mitochondrial targeting mechanism

Markus Wild[1], Friedrich Hahn[1*], Benedikt Grau[2], Lars Herrmann[2], Aischa Niesar[1], Martin Schütz[1], Mélanie M. Lorion[3], Lutz Ackermann[3], Svetlana B. Tsogoeva[2], Manfred Marschall[1,*]

[1] Institute for Clinical and Molecular Virology, Friedrich-Alexander University of Erlangen-Nürnberg (FAU), Schlossgarten 4, 91054 Erlangen, Germany; markus.wild@uk-erlangen.de, friedrich.hahn@uk-erlangen.de, aischa.niesar@uk-erlangen.de, manfred.marschall@fau.de

[2] Institute of Organic Chemistry I, FAU, Nikolaus-Fiebiger-Straße 10, 91058 Erlangen Germany; benedikt.grau@fau.de, lars.herrmann@fau.de, svetlana.tsogoeva@fau.de

[3] Institute for Organic and Biomolecular Chemistry, Georg-August-Universität Göttingen, Tammannstraße 2, 37077 Göttingen, Germany; melanie.lorion@chemie.uni-goettingen.de, lutz.ackermann@chemie.uni-goettingen.de

* Correspondence: manfred.marschall@fau.de (M.M.), friedrich.hahn@uk-erlangen.de (F.H.); Tel.: ++49 9131 8526089 (M.M.), ++49 9131 8536480

Table S1. Characteristics of mitochondria under treatment with BG95/ART compounds in HFFs and U373 cells.[#]

Cell type	Occurrance of BG95 bodies	Colocalization of BG95 bodies with prohibitin	HCMV-induced dispersed mitochondria	BG95-induced punctate mitochondria	BG95-related compounds also induce punctate mitochondria
HFF	+	+	+	+	+
U373	+	+	nd*	nd*	nd*

[#] For evaluation of characteristics see Figures 2-5, 7 and S3-S5.
* nd, not determined

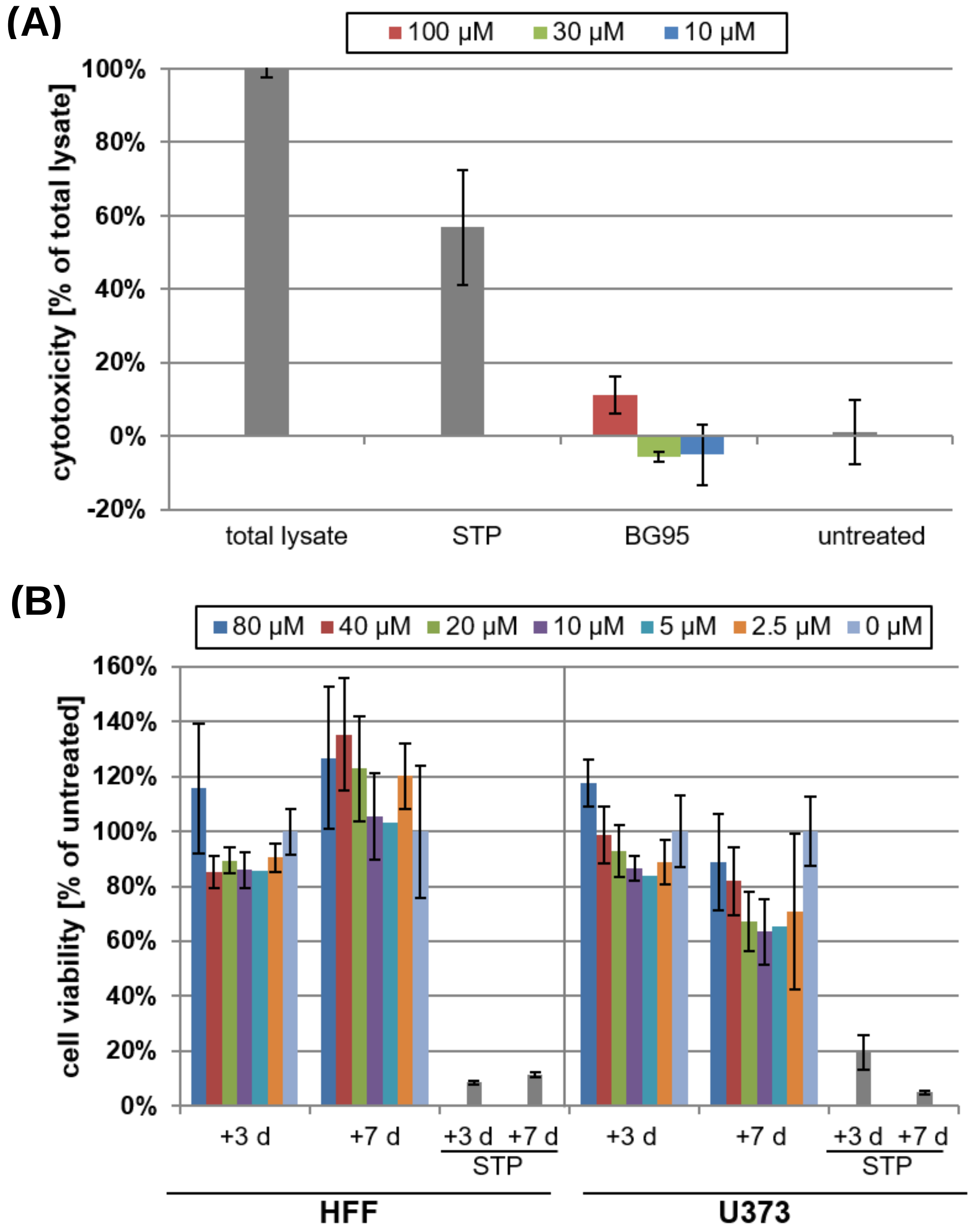

Figure S1. BG95 shows no cytotoxic activity after incubation periods of 1, 3 and 7 d. **(A)** Lactate dehydrogenase release assay was performed under standard conditions using cell culture media supernatants of HFFs treated with the indicated concentrations of BG95 for 1 d. As a positive control, the cytotoxicity-inducing kinase inhibitor staurosporine (STP, 0.1 µM) was used. **(B)** Neutral red cytotoxicity assay was performed on HFF or U373 cell layers treated with the indicated concentrations of BG95 for 3 d and 7 d, using 1 µM of STP as a positive control.

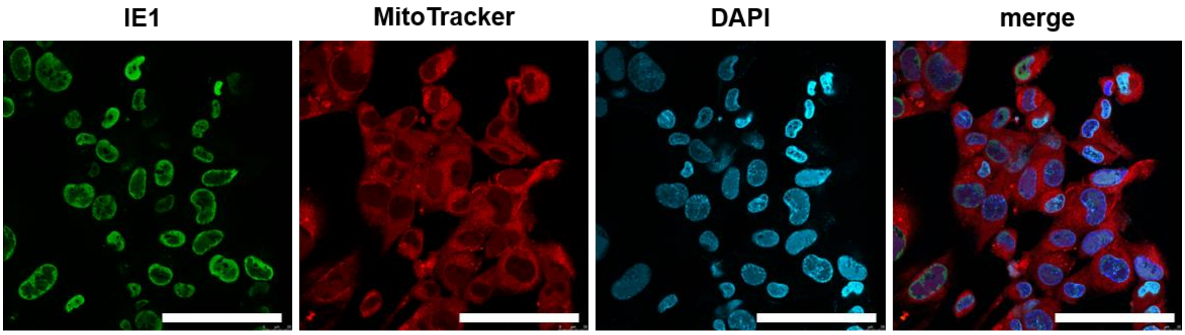

Figure S2. Productive infection of HFFs with HCMV strain AD169 was visualized by the detection of IE1 expression. HFFs were infected at an MOI of 2, fixed 4 d p.i. and IE1 expression detected by immunofluorescence staining using MAb-IE1 antibody (p63-27). Scale bars represent 100 µm.

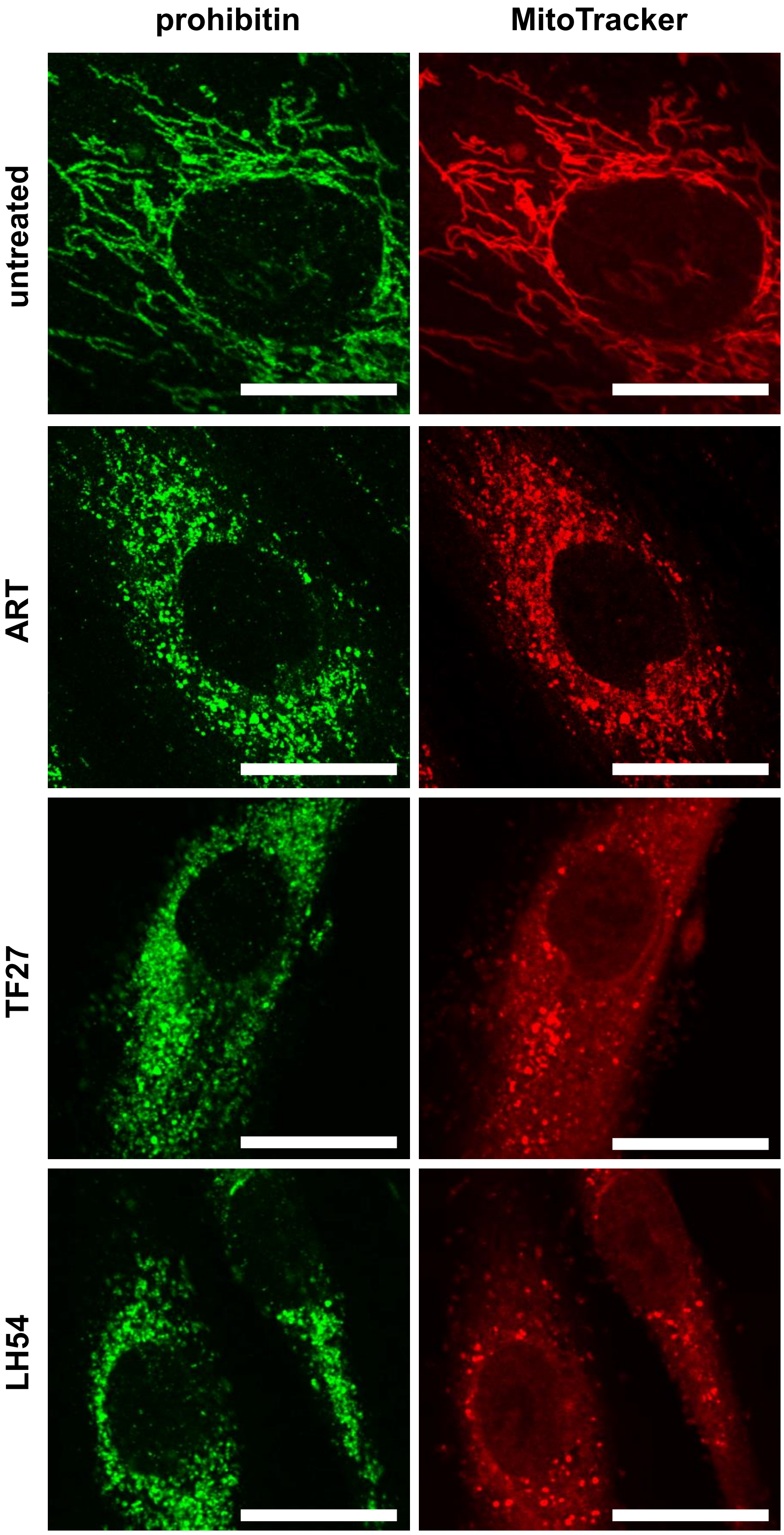

Figure S3. Artesunate and its derivatives TF27 and LH54 elicit changes to mitochondrial architecture of HFFs. Loss of the characteristic filamentous structure and fragmentation of mitochondria into a punctate phenotype was observed upon treatment with 10 μM of ART, TF27 and LH54, similar to those induced by BG95 treatment. Scale bars represent 25 μm.

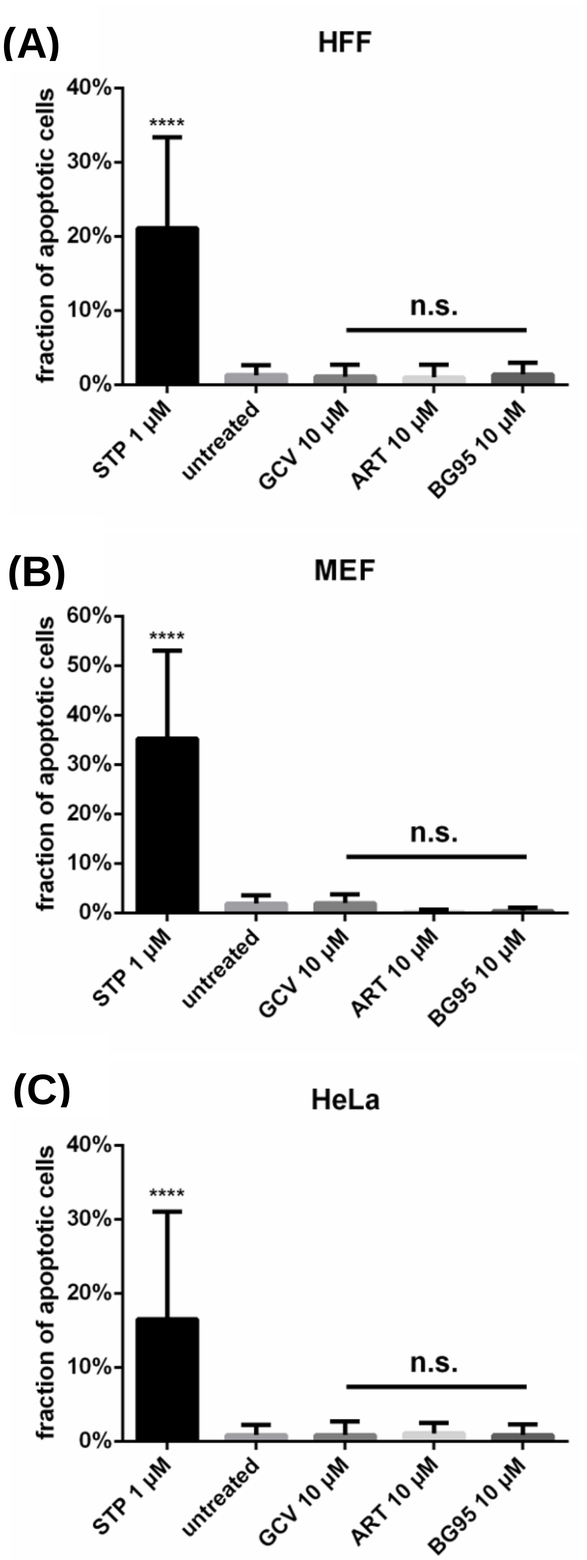

Figure S4. BG95 does not induce apoptosis in primary human or murine fibroblasts, i.e., HFFs **(A)** or MEFs **(B)**, and HeLa cells **(C)**. Cells were treated with the indicated concentrations of compounds or remained untreated for 3 d, after which they were subjected to NucView staining, fixation and DAPI staining. The fraction of NucView-positive cells was counted in at least 20 pictures per compound. Data is given as the mean of the fractions of individual pictures + SD. STP was used as a positive control (1 µM, added 4 h before fixation). Statistical analysis was performed using ordinary One-way ANOVA followed by post-hoc Tukey's test compared to untreated. ****, $p < 0.0001$; n.s., not significant.

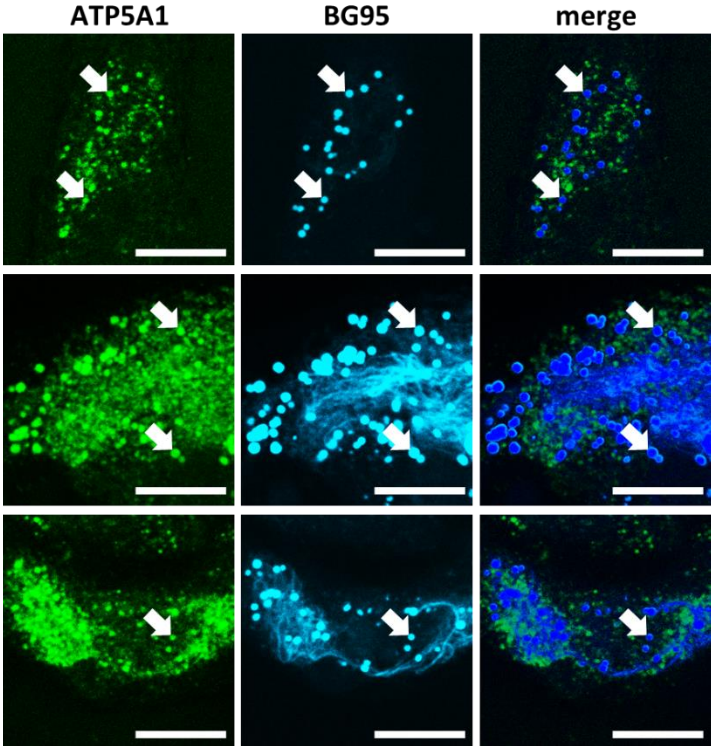

Figure S5. Autofluorescent BG95 accumulates in characteristic intramitochondrial bodies as indicated by the ATP5A1 marker protein. HFFs were infected with HCMV AD169 (MOI 2), treated with 10 μM of BG95, fixed at 3 d p.i. and immunostained using an ATP5A1-specific antibody (7H10BD4F9, Invitrogen). At 3 d p.i., BG95 bodies colocalized with mitochondrial marker ATP5A1 (similar to prohibitin, see Figs. 2, 3 and 5). White arrows indicate BG95 bodies, referring to the corresponding positions in other channels. Note the appearance of additional, so far uncharacterized thread-like autofluorescent structures particularly in the lower two panels. Scale bars represent 20 μm.
